# The E3 ubiquitin ligase Itch regulates death receptor and cholesterol trafficking to affect TRAIL-mediated apoptosis

**DOI:** 10.1038/s41419-023-06417-4

**Published:** 2024-01-12

**Authors:** James Holloway, Aidan Seeley, Neville Cobbe, Richard C. Turkington, Daniel B. Longley, Emma Evergren

**Affiliations:** Patrick G Johnston Centre for Cancer Research, Queen’s University Belfast, 97 Lisburn Road, BT9 7AE Belfast, UK

**Keywords:** Apoptosis, Ubiquitylation

## Abstract

The activation of apoptosis signalling by TRAIL (TNF-related apoptosis-inducing ligand) through receptor binding is a fundamental mechanism of cell death induction and is often perturbed in cancer cells to enhance their cell survival and treatment resistance. Ubiquitination plays an important role in the regulation of TRAIL-mediated apoptosis, and here we investigate the role of the E3 ubiquitin ligase Itch in TRAIL-mediated apoptosis in oesophageal cancer cells. Knockdown of Itch expression results in resistance to TRAIL-induced apoptosis, caspase-8 activation, Bid cleavage and also promotes cisplatin resistance. Whilst the assembly of the death-inducing signalling complex (DISC) at the plasma membrane is not perturbed relative to the control, TRAIL-R2 is mis-localised in the Itch-knockdown cells. Further, we observe significant changes to mitochondrial morphology alongside an increased cholesterol content. Mitochondrial cholesterol is recognised as an important anti-apoptotic agent in cancer. Cells treated with a drug that increases mitochondrial cholesterol levels, U18666A, shows a protection from TRAIL-induced apoptosis, reduced caspase-8 activation, Bid cleavage and cisplatin resistance. We demonstrate that Itch knockdown cells are less sensitive to a Bcl-2 inhibitor, show impaired activation of Bax, cytochrome c release and an enhanced stability of the cholesterol transfer protein STARD1. We identify a novel protein complex composed of Itch, the mitochondrial protein VDAC2 and STARD1. We propose a mechanism where Itch regulates the stability of STARD1. An increase in STARD1 expression enhances cholesterol import to mitochondria, which inhibits Bax activation and cytochrome c release. Many cancer types display high mitochondrial cholesterol levels, and oesophageal adenocarcinoma tumours show a correlation between chemotherapy resistance and STARD1 expression which is supported by our findings. This establishes an important role for Itch in regulation of extrinsic and intrinsic apoptosis, mitochondrial cholesterol levels and provides insight to mechanisms that contribute to TRAIL, Bcl-2 inhibitor and cisplatin resistance in cancer cells.

## Introduction

Itch (AIP4) is a member of the of E3 ubiquitin ligase family that mediates lysine-29, -48 and -63-linked ubiquitin conjugation [1]. It is associated with pro-proliferative activities and endocytic trafficking [2]. Itch is composed of an N-terminal lipid binding C2 domain, a proline rich region, WW motifs and in the C terminus a HECT (homologous to the E6-AP C-terminus) domain. The two WW motifs mediate an intramolecular interaction that keeps Itch in an inactive conformation. Several substrates have been identified for Itch; including FLIP, Numb, SREBP2, chemokine receptor CXCR4 and Bid [3]. Regulation of the anti-apoptotic protein FLIP is important during cell death, and it has been shown that ubiquitination by Itch promotes its degradation and reduces its protein levels [4, 5]. Itch also interacts with and ubiquitinates endocytic proteins including endophilin, intersectin-1, amphiphysin, Snx9, and Pacsin, which results in regulation of trafficking of cell surface receptors [6]. Itch is highly expressed in the gastrointestinal tract, brain and skeletal muscle, and it is overexpressed in multiple types of cancers [7].

The regulation of death receptor signalling by ubiquitination has recently emerged as an important mechanism of resistance to cell death in cancer cells [8, 9]. Core components of the TRAIL signalling pathway have been demonstrated to be regulated by ubiquitination, namely TRAIL-R1/2, FLIP and caspase-8. To evade cell death cancer cells frequently use the ubiquitin-proteasomal system for degradation of pro-apoptotic proteins [10, 11]. TRAIL-mediated apoptosis is unique because it selectively induces apoptosis in cancer cells compared to untransformed cells [12–14]. Binding of the ligand TRAIL (TNF-related apoptosis-inducing ligand) to death receptors (TRAIL-R1, TRAIL-R2) on the cell surface results in recruitment of FADD and activation of procaspase-8 or -10 and the subsequent cleavage of Bid, which results in its translocation to mitochondria and initiation of BAX/BAK pore formation. Bid is a key mediator of both intrinsic and extrinsic apoptosis and has been proposed to define whether TRAIL-mediated apoptosis is mitochondria-dependent or -independent [15, 16]. A regulator of caspase-8 activity, and Bid cleavage, is the FLICE-like inhibitory protein (FLIP); FLIP is a procaspase-8 homologue that can compete with caspase-8 for binding to FADD and is itself negatively regulated by ubiquitination [3, 5].

We hypothesized that Itch may play a role in the regulation of TRAIL-induced apoptosis by altering the abundance and/or subcellular distribution of FLIP or TRAIL receptors. In the TCGA pan-cancer atlas the three cancer types with the highest gain of copy number for ITCH were rectum, colon and oesophageal carcinoma making these interesting disease settings in which to study Itch. Consequently, we compared the expression of ITCH in cell lines from these cancers and focussed our work on the cell line with the highest expression of ITCH, OE33, an oesophageal cell line [17]. In this study, we examined whether knockdown of Itch affects the endocytosis of TRAIL-R2/R1, FLIP expression and TRAIL-mediated signalling in the oesophageal cell line OE33 that displays a high expression of Itch. We demonstrate that the E3 ubiquitin ligase Itch regulates TRAIL-mediated cell death and trafficking of the TRAIL-R2 receptor, cholesterol trafficking to mitochondria, Bax activation and cytochrome c release. We propose a mechanism where Itch regulates import of anti-apoptotic mitochondrial cholesterol through interaction with a lipid transfer protein complex.

## Materials and methods

### Cell Lines

OE33, LIM1215 and the KM12 cell lines were maintained in RPMI media (Gibco, UK). COLO320, HCT116 and HT29 cell lines were maintained in Macoy’s5A media (Gibco, UK). Cell culture media was supplemented with 10% foetal bovine serum (Gibco, UK), 5% Penicillin/Streptomycin (Gibco, UK) and 2mM L-glutamine (Gibco, UK). All cells were maintained at 37 °C in a 5% CO_2_ humidified atmosphere and were regularly screened for the presence of mycoplasma using the MycoAlert Mycoplasma Detection Kit (Lonza, Switzerland).

### Reagents and antibodies

The following commercial antibodies were used: TRAIL-R2/DR5 (rabbit, Cat# 3696 S, Cell Signalling Technology); BID (rabbit; Cat# 2002S, Cell Signalling Technology); PARP (rabbit, Cat# 9542 S, Cell Signalling Technology); Caspase 3 (rabbit, Cat# 9662 S, Cell Signalling Technology); FLIP NF6 (mouse, Cat# AG-20B-0056-C100, Adipogen); Itch (mouse, Cat# 611198, BD Biosciences); FADD (mouse, Cat# 556402, BD Pharmigen); Caspase-8 (mouse, Cat# ALX-804-242-C100, Enzo); TRAIL-R1/DR4 (rabbit, Cat# AB16955, Calbiochem); SREBP2 (rabbit, Cat# ab30682, Abcam); beta-Actin (mouse, Cat# A5316, Sigma), cytochrome C (BD Biosciences, # 556433), BAX 6A7 (Thermo #MA5-14003), StAR (#8449; Cell Signalling Technology), VDAC2 (ab155803; AbCam), Bax (#2772; Cell Signalling Technology) and GAPDH (mouse, Cat# sc-47724, Santa Cruz). The following PE-conjugated antibodies were used in cell surface expression experiments: isotype control (Cat# 12-4714-73, eBioscience); DR5 (Cat# 12-9908-42, eBioscience) and DR4 (Cat# 12-6644-42, eBioscience). AMG655 (Conatumumab) was sourced from Amgen Inc (Thousand Oaks, CA, USA); this antibody was coupled to Dynabeads using the Dynabead Coupling Kit (Life Technologies, Paisley, UK) for use in TRAIL-R2 DISC IP assay. The secondary antibodies used for immunocytochemistry were Alexa Fluor488- and 568- conjugated goat anti-rabbit, anti-mouse and anti-sheep (Thermo Fischer Scientific); for Western blot detection goat anti-rabbit and anti-mouse IgG- HRP conjugates (Cell Signalling Technologies). The following reagents were used: Filipin III (SAE0087; Sigma-Aldrich); DAPI (#62248; Thermo Fischer Scientific); U18666A (#662015; Calbiochem); ABT-263 (#S10001; Selleckchem); Alexa488-wheat germ agglutinin (#W11261; Thermo Fischer Scientific). Human isoleucine-zipper TRAIL (izTRAIL) was expressed and purified as described and stored at -80 °C in aliquots [18]. The smartpool siRNA (Dharmacon, USA) for ITCH were; GUUGGGAACUGCUGCAUUA, CAACAUGGGACGUAUUUAU, GAAAUUAAGAGUCAUGAUC and CGAAGACGUUUGUGGGUGA.

### Knockdown cell line generation

To generate *ITCH* knockdown cell lines lentivirus expressing short hairpin RNA (shRNA) from the pLKO1.puro plasmid were generated with the targeting sequence of the *ITCH* gene AACACCTCGAGACAACCTC (shEE162) and the shRNA control CAACAAGATGAAGAGCACCAA (Sigma MISSION Target shRNA). Selection was carried out using 1 µg/ml of puromycin for 48 h. The efficiency of shRNA knockdown was assessed by Western blot.

### Immunoblotting

Whole cell lysate was prepared in RIPA buffer and Western blot analysis was carried out as described previously [19]. Protein expression was detected using the Western Lighting Plus-ECL substrate (PerkinElmer, Waltham, MA) on a G:BOX Chemi6 gel doc system (Syngene). Densitometry was carried out using ImageJ software. Uncropped blots are shown in Supplementary Fig. 4.

### Flow cytometry assays

To assess cell death, live cell staining with FITC-tagged AnnexinV (BD Biosciences) and addition of PI (Sigma) were analysed on a BD Accuri C6 Plus flow cytometer (BD Biosciences). Cell surface TRAIL-R1 and TRAIL-R2 expression was assessed following live cell staining with Phycoerythrin-conjugated antibodies. All experiments were gated using an isotype control antibody.

### Cell viability assay

Cell viability was assessed using CellTiterGlo Luminescent Assay (Promega, Madison, WI) according to manufacturer’s instructions on a Biotek plate reader.

### Caspase activity assay

Caspase activity was assessed using Caspase-3/-7 Glo Luminescent Assay (Promega, Madison, WI) according to manufacturer’s instructions on a Biotek plate reader.

### Cytochrome C release assay

The assay was essentially performed as previously described [20, 21]. Cells were collected, washed in PBS, resuspended in ice cold permeabilization buffer (150 mM KCl, 1 mM EDTA, 200 μg/ml digitonin, in PBS) and incubated on ice for 5 min. Cells were washed once in PBS, fixed in 4% paraformaldehyde at room temperature for 20 minutes, washed 3 times in PBS (700 × *g* for 5 min at 4 °C) and resuspended in blocking buffer (2% BSA in PBS) for 30 min at room temperature. Cells were incubated with anti-cytochrome C antibodies at 1:200 dilution in blocking buffer overnight at 4 °C followed by Alexa-488 anti–mouse IgG antibody at a 1:200 dilution in blocking buffer for 1 h. Following washing 3 times in blocking buffer the samples were analysed on a BD Accuri C6 Plus flow cytometer (BD Biosciences) equipped with the BD CSampler Plus software (BD Biosciences).

### BAX IP

Roughly 10 × 10^6^ cells were used per sample. Cells were treated for 6 h with 50 ng/ml izTRAIL, lysed in 100 μl CHAPS buffer (150 mM sodium chloride, 10 mM HEPES pH 7.4, 1.0% CHAPS) with protease inhibitors for 1 hour on ice. The lysate was centrifuged at 15,000 × *g* for 20 min. Each sample contained 500 µg of protein in of 500 µl lysis buffer with 2 μg of BAX 6A7 antibody. The samples were incubated at 4 °C with rotation overnight. 25 μl of protein A agarose beads were preincubated with 500 µl of CHAPS buffer at 4 °C with rotation for 3 h, and then added to the samples for 30 min. Immunoprecipitates were collected via a brief spin (2500 × *g* for 5 min at 4 °C), washed four times with 500 µl of CHAPS lysis buffer (with 0.2% NP-40 buffer), and then solubilized with 2× SDS–PAGE sample buffer.

### DISC-IP

The TRAIL-R2 DISC-IP was carried out as previously described and recruitment of DISC proteins was assessed by Western blotting [22].

### Immunofluorescence

Immunocytochemistry was performed as described previously [19]. Confocal images were acquired of the cells at room temperature using a Leica SP8 confocal microscope equipped with a 63x objective (1.4 NA HCX PL APO lens) controlled by the Leica Application Suite-X software.

### Image analysis

Confocal image analysis was carried out using the ImageJ software. Receptor expression was expressed as the Integrated Density of the stain at the plasma membrane divided by the area. The plasma membrane was identified using wheat germ agglutin stain and the integrated density was measured by highlighting the intensity of the receptor staining at the plasma membrane. Colocalization analysis was performed using the Pearson colocalization coefficient using the JaCOP plugin.

### Transmission electron microscopy

Embedding and processing of samples was performed as described [19]. Images were acquired on a Jeol JEM1400plus microscope at 80 kV equipped with a JEOL Ruby (8 MPixel) bottom-mounted CCD camera.

### Statistical data analysis

Statistical significance was calculated from distinct technical replicates (*n* ≥ 3; unless otherwise stated) and confirmed in at least three independent experiments either by Student’s T test (two-tailed) or two-way ANOVA in GraphPad Prism 9. Two-way ANOVA tests were performed to compare two factors (for e.g. shRNA and drug concentration). The Student’s *t* test was used for comparison of data with a normal distribution and equal variance. Outliers were identified by a ROUT test. Graphs were plotted as means with error bars represented as standard error of mean; statistical significance was denoted as follows: ***p < 0.001, **p < 0.01, *p < 0.05, ns = *p* > 0.05.

## Results

### Itch regulates TRAIL-mediated apoptosis independently of FLIP

**Fig. 1:**
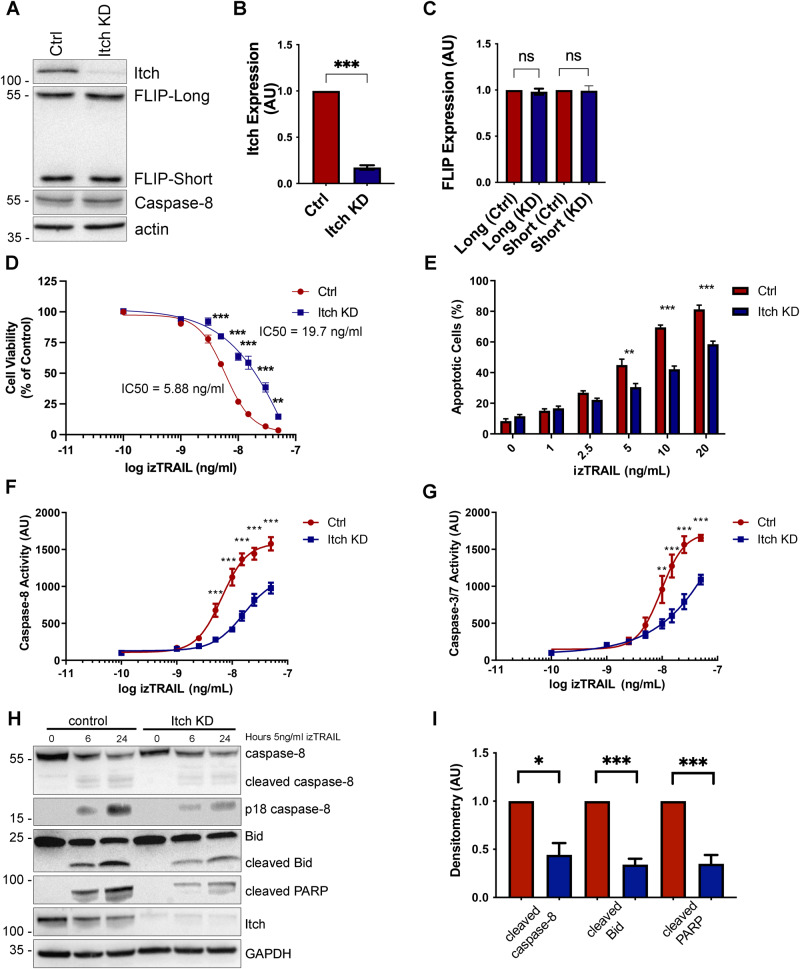
Itch knockdown increases resistance to TRAIL-mediated apoptosis in OE33 cells and impairs caspase-8 cleavage and activity. **A** Western blot analysis of basal expression of FLIP(L), FLIP(S), FADD, Procaspase-8, TRAIL-R1, TRAIL-R2 in OE33 Ctrl (shCTRL) and Itch knockdown (KD) cells stably expressing shRNA targeting ITCH. **B** Bar graph showing the densitometry of Western blot analysis of Itch expression normalised to the actin loading control in five independent experiments. **C** Bar graph showing the quantification for FLIP-Long and -Short expression in control shRNA and Itch knockdown (KD) cell lines. *N* = 5 independent experiments. **D** OE33 Ctrl and Itch KD cell lines were subjected to treatment with increasing doses of izTRAIL for 24 h (0–50 ng/mL). Cell viability was measured using the CellTiterGlo Assay. Data was normalised to an untreated control. **E** OE33 Ctrl and Itch KD cells were treated with 0–20 ng/mL TRAIL for 24 h and then stained with FITC-conjugated Annexin V and propidium iodide to assess the number of apoptotic cells under each treatment condition. Bar graph showing the percentage of apoptotic cells (Annexin V-positive cells) of the population. **F** Caspase -8 and (**G**) Caspase -3/-7 activity in OE33 Ctrl and Itch KD cell lines was measured following treatment with increasing concentrations of izTRAIL (0–50 ng/mL). Activity was measured 6 h post treatment using the Caspase -3/-7 or the Caspase -8 Glo assays. **H** Western blot analysis of cleaved Caspase-8, Bid and PARP in OE33 Ctrl and Itch KD cell lines following treatment for 6 or 24 h with 5 ng/ml izTRAIL. **I** Densitometry of the experiment in (**C**) in four independent repeats. Error bars represent the standard error of the mean. Statistical significance was measured by Student’s *t* test. *p < 0.05, **p < 0.01, ***p < 0.001.

To investigate the role of Itch in TRAIL-mediated apoptosis, we generated a stable knockdown in the oesophageal cancer cell line OE33 and subjected it to TRAIL treatment followed by quantification of cell viability and apoptosis. Western blot analysis demonstrated a reduction in protein expression of 80% in the OE33 Itch knockdown (KD) cell line with no change in the expression of pro-caspase-8 or the two FLIP splice forms (Fig. 1A–C). A similar lack of impact on FLIP expression was observed in a panel of oesophageal and colorectal cancer cell lines (Supplementary Fig. 1). FLIP(L) has been shown to be ubiquitinated by Itch in a few cancer cell lines and fibroblasts, while in macrophages its stability is not directly regulated by Itch [5, 23–26].

To assess the functional effects of Itch knockdown on TRAIL-induced apoptosis, we used recombinant isoleucine-zipper (iz) TRAIL, which maintains the trimeric nature of the cell surface TRAIL ligand and enhances its activity [27]. The Itch KD cell line was significantly more resistant to the izTRAIL-induced apoptosis as assessed by cell viability (*p* < 0.001; Fig. 1D) and apoptosis assays (*p* < 0.001; Fig. 1E). The Itch KD cell line also displayed significantly reduced activity of caspase-8 (*p* < 0.001; Fig. 1F) and caspase-3/7 (*p* < 0.001; Fig. 1G) following treatment with izTRAIL. These results were corroborated by a reduction of cleaved caspase-8, PARP and Bid in the Itch KD cell line (Fig. 1H, I**;** *p* < 0.001). In conclusion, our findings demonstrate that Itch regulates TRAIL-mediated apoptosis through a mechanism that is not dependent on FLIP.

### Itch knockdown causes a mis-localization of TRAIL-R2 in OE33 cells

**Fig. 2:**
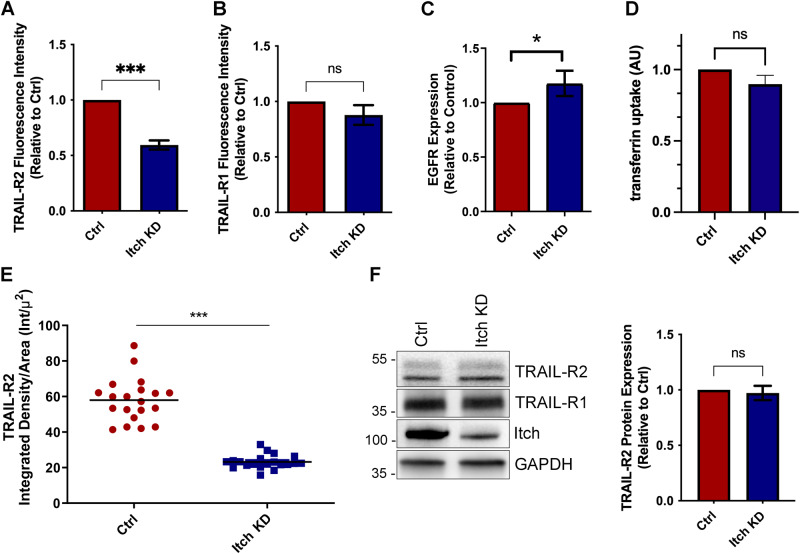
Itch knockdown cells show reduced surface expression of TRAIL-R2 in OE33 cells. FACS analysis of cell surface expression of (**A**) TRAIL-R2 and (**B**) TRAIL-R1 cell surface expression in OE33 Ctrl and Itch KD cell lines. Bar graphs showing the mean fluorescence intensity of cell surface staining of TRAIL-R2 in OE33 control and Itch KD cells in three independent experiments (*n* = 10,000 cells per experiment). **C** FACS analysis of cell surface expression of EGFR in serum starved cells normalised to the control cell line in three independent experiments. **D** FACS analysis of fluorescent transferrin uptake in serum starved cells. Three independent experiments were performed where 10,000 cells were analysed per experiment. **E** TRAIL-R2 cell surface expression was measured using confocal microscopy. Alexa488-tagged wheat germ agglutinin stain was used to identify the plasma membrane and anti-DR5 antibody stain was used to determine the intensity of TRAIL-R2 at the plasma membrane. Receptor expression is expressed as the Integrated Density of the stain at the plasma membrane divided by the area. *N* = 20. **F** Western blot showing the total expression levels of TRAIL-R2 and -R1 in control and Itch KD OE33 cells. Error bars represent the standard error of the mean. Statistical significance was calculated by Student’s *t* test; ns, not significant, *p < 0.05, ***p < 0.001.

Next, we sought to investigate whether loss of Itch mediates TRAIL resistance through downregulation of the cell surface expression of death receptors. A major limiting factor of apoptotic TRAIL signalling is the availability of the receptor at the cell surface [28, 29]. Itch targets components of the endocytic machinery and regulates signalling and stability of cell surface receptors [30]. A significant reduction in cell surface TRAIL-R2 expression was observed in the Itch knockdown compared to the control (Fig. 2A; *p* < 0.001). In comparison, the cell surface expression of TRAIL-R1 (DR4) was found to be unaltered between the two cell lines (Fig. 2B), while Epidermal Growth Factor receptor (EGFR) was increased (*p* < 0.05; Fig. 2C) and transferrin internalisation was unchanged (Fig. 2D). TRAIL-R1, -R2 and EGFR are internalised after ligand binding by both clathrin-dependent and -independent endocytosis while the transferrin receptor is only internalised by clathrin-mediated endocytosis [31]. Therefore, these data suggested that clathrin-mediated receptor endocytosis was not enhanced by Itch depletion and altered receptor trafficking could not explain the reduction of TRAIL-R2 on the cell surface. Immunocytochemistry supported the finding that TRAIL-R2 cell surface expression was reduced in the Itch KD OE33 cell line (Fig. 2E). Importantly, a loss of Itch expression did not impact on total TRAIL-R1/R2 receptor (DR4/5) protein expression (Fig. 2F). To validate these findings further, a panel of oesophageal and colorectal cancer cell lines were subjected to Itch knockdown, which resulted in a reduced TRAIL-R2 cell surface expression (Supplementary Fig. 2). In summary, knockdown of Itch resulted in a resistance to TRAIL-mediated apoptosis and correlated with a loss of TRAIL-R2 at the plasma membrane but not TRAIL-R1. This indicated that Itch either regulates anterograde membrane trafficking of TRAIL-R2 (but not TRAIL-R1), enhances the assembly of the DISC complex, or regulates association of caspase-8 and/or Bid with mitochondria. The first option seemed unlikely as there are no data suggesting that the two TRAIL receptors traffic to the plasma membrane via independent mechanisms and we therefore focussed on exploring the other two options.

### TRAIL-activated DISC assembly is independent of Itch

**Fig. 3:**
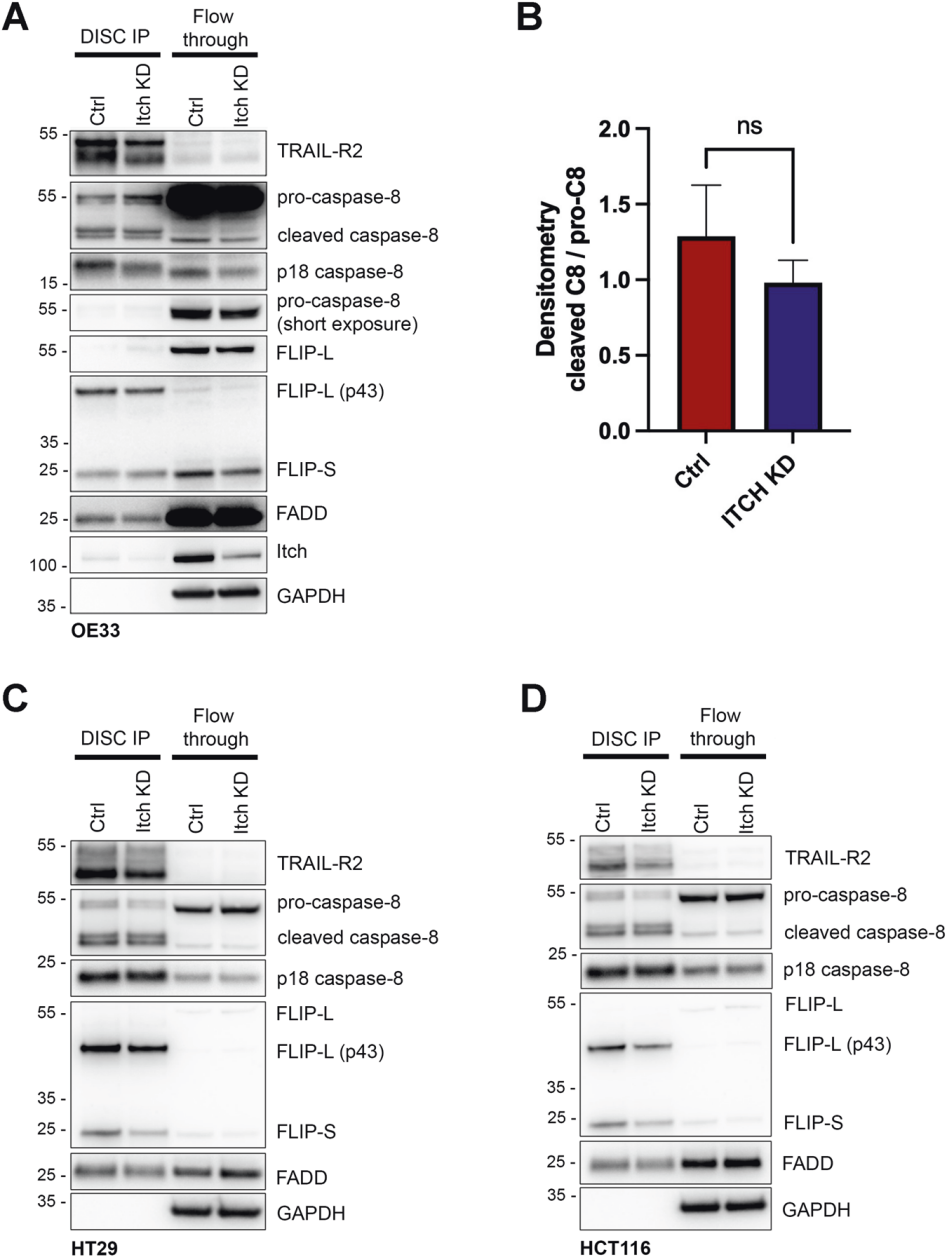
Itch is found at the death-inducing signalling complex (DISC) but does not regulate its composition or cleavage of caspase-8. **A** Immunoprecipitation of the DISC using an activating TRAIL-R2 antibody from OE33 cells with a stable Itch knockdown (KD) or a control. Cells were treated for 1 h to induce formation of the DISC. Proteins were separated by size on a SDS-PAGE gel prior to Western blot analysis. **B** Densitometry of cleaved caspase-8 normalised to the pro-caspase-8 in the DISC IP from OE33 cells from three independent experiments. **C** Immunoprecipitation of the DISC in HT29 cells treated with control or ITCH siRNA using the TRAIL-R2 antibody. **D** Immunoprecipitation of the DISC in HCT116 cells treated with control or ITCH siRNA using the TRAIL-R2 antibody. Statistical significance was measured by Student’s *t* test, ns represents a non-significant difference.

We hypothesised that Itch may be incorporated into the death receptor signalling complex (DISC) where it could regulate caspase-8 recruitment or activation. Next, we investigated whether Itch regulates the assembly of the DISC. A reduction in TRAIL-R2 recruitment into the DISC in the Itch KD cells was observed (Fig. 3A), consistent with reduced cell surface levels of TRAIL-R2 (Fig. 2A). However, this did not correlate with significantly reduced recruitment of caspase-8, FADD or FLIP(L). Furthermore, caspase-8 dependent cleavage of FLIP-L at the DISC was maintained in the OE33 Itch KD cell line, and all other components of the complex are there in equal amounts to the control suggesting that Itch does not regulate the DISC assembly or activation of caspase-8 at the DISC (Fig. 3A, B). Similar results were observed in the HT29 and HCT116 cell lines (Fig. 3C, D). Taken together, our findings indicate that Itch regulation of caspase-8 activity and apoptosis is not due to alterations in the death receptor complex at the plasma membrane.

### Itch knockdown regulates mitochondrial cholesterol content which implicates a novel regulatory role in apoptosis

Based on our findings showing that receptor endocytosis was not significantly altered in the Itch KD cell line and the assembly of the DISC and recruitment of caspase-8 was not impaired we next investigated the role of mitochondria in apoptosis in these cells. The OE33 cell line is classified as a type II cell reliant on the intrinsic apoptotic pathway. The lipid composition of the mitochondrial outer membrane is an important regulator of caspase-8 recruitment, activity [32, 33], and subsequent cleavage of Bid to induce apoptosis [34]. Furthermore, mitochondrial cholesterol mitigates against apoptosis by increasing the stiffness of the mitochondrial membrane to prevent perforations and release of cytochrome c [33], which is significant as Itch regulates cholesterol metabolism through ubiquitination of nuclear SREBP2 and SIRT6 resulting in increased intracellular cholesterol levels [35]. We therefore investigated whether a defect in cholesterol homoeostasis in the Itch KD OE33 cell line could affect mitochondrial cholesterol content, caspase-8 activity and cell viability.

**Fig. 4:**
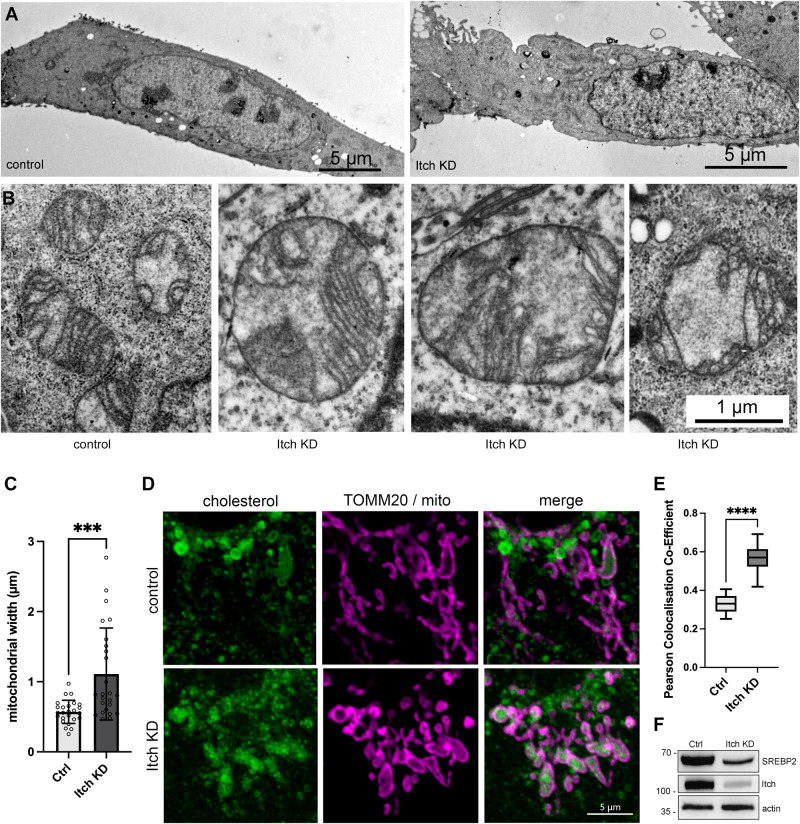
Itch knockdown leads to an accumulation of mitochondrial cholesterol. **A** Transmission electron micrographs illustrating the morphology of control and Itch KD cells. Scale bars, 5 µm. **B** Electron micrographs illustrating the morphology and size of mitochondria in control and Itch KD cells. Scale bar, 1 µm for all images. **C** Quantification of mitochondrial width from 25 cells. Mean ± SD. **D** Confocal images illustrating the accumulation of free cholesterol by Filipin-III staining together with outer mitochondrial membrane marker TOM20. Scale bar, 5 µm. **E** Tukey box plot displaying the Pearson colocalization co-efficient of TOM20 and Filipin staining to compare levels of mitochondrial cholesterol in control and Itch KD cell lines. *N* = 14 per group. **F** Western blot analysis of cell lysates showing the expression of nuclear SREBP2 (cleaved) protein in the Itch KD compared to control. Statistical significance was measured by Student’s *t* test. ***p < 0.001, ****p < 0.0001.

While the morphology of the endoplasmic reticulum (ER) and Golgi appeared normal, the morphology of the mitochondria was significantly altered in the KD (Fig. 4A, B). Control mitochondria had a normal oblong shape, while mitochondria in the Itch KD cells were often round and significantly wider (*p* < 0.001; Fig. 4C). Furthermore, disorganised cristae were observed indicating an accumulation of cholesterol in mitochondria [36]. To investigate whether there was an increase in mitochondrial cholesterol in Itch KD cells they were stained with Filipin-III dye (Fig. 4D). An increase in Filipin-III staining of mitochondria was observed in OE33 Itch KD and this observation was corroborated in HT29 and HCT116 cells (Supplementary Fig. 3). A significant increase in colocalization of mitochondrial outer membrane with the Filipin-cholesterol stain was observed in the KD compared to control (*p* < 0.0001; Fig. 4E). The images further illustrate the altered morphology of mitochondria in cells with reduced Itch expression. Finally, we investigated whether SREBP2 expression was altered in the OE33 knockdown cell line as it is a main regulator of cholesterol metabolism. When cholesterol levels are low SREBP2 is proteolytically activated and translocated to the nucleus to regulate transcription of genes involved in cholesterol synthesis and uptake. A reduction in the mature nuclear form of SREBP2 was observed in the knockdown compared to the control indirectly indicating elevated cholesterol levels (Fig. 4F). In summary, the data show that Itch regulates cholesterol content in mitochondria.

### Accumulation of cholesterol in mitochondria cause resistance to TRAIL treatment and inhibits caspase-8 cleavage

**Fig. 5:**
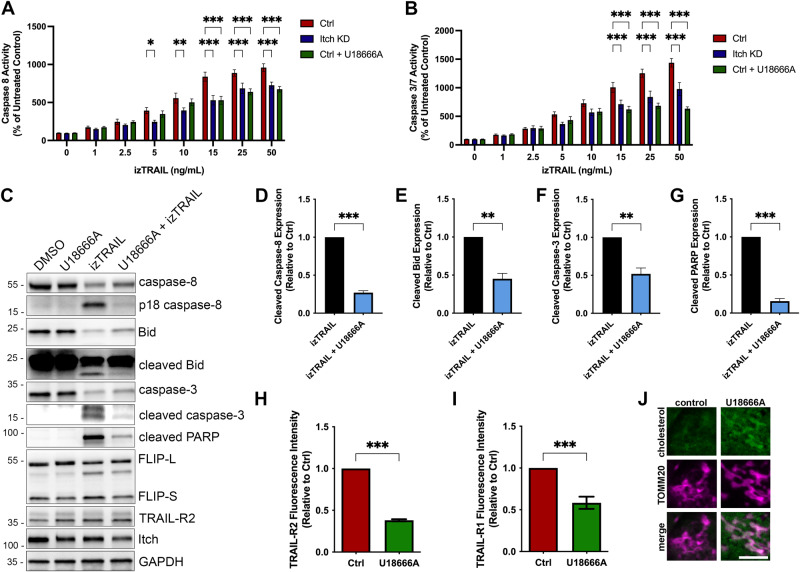
Cholesterol homoeostasis is an important resistance mechanism for TRAIL-induced apoptosis. **A** Caspase-8 activity and (**B**) Caspase-3/-7 activity was measured following treatment with increasing concentrations of izTRAIL (0–50 ng/mL). Activity was measured 6 h post treatment using the Caspase-3/-7 or the Caspase-8 Glo assays. Cells were treated with U18666A (1 ng/ml) for 24 h. **C** Western blot analysis of cell lysates after treatment with U18666A and izTRAIL for key apoptotic proteins; caspase-8, -3, Bid and PARP. **D–G** Quantification of an inhibition of apoptosis in OE33 cells treated with U18666A (1 ng/ml) in combination with izTRAIL, showing a significant loss of cleavage of caspase-8, -3, Bid, and PARP in three independent experiments. **H** Bar graph showing the mean fluorescence of cell surface expression of TRAIL-R2 stained control and cells treated with U18666A in three independent experiments. **I** Bar graph showing the mean fluorescence intensity of cell surface expression of TRAIL-R1 in control and U18666A-treated (1 ng/ml) OE33 cells in three independent experiments (*n* = 10,000 cells per experiment). **J** Fluorescence images of OE33 cells showing the accumulation of cholesterol, stained with Filipin-III, in mitochondria after U18666A treatment. Scale bar, 5 µm. Statistical significance was measured by Student’s *t* test. *p < 0.05, **p < 0.01, ***p < 0.001.

**Fig. 6:**
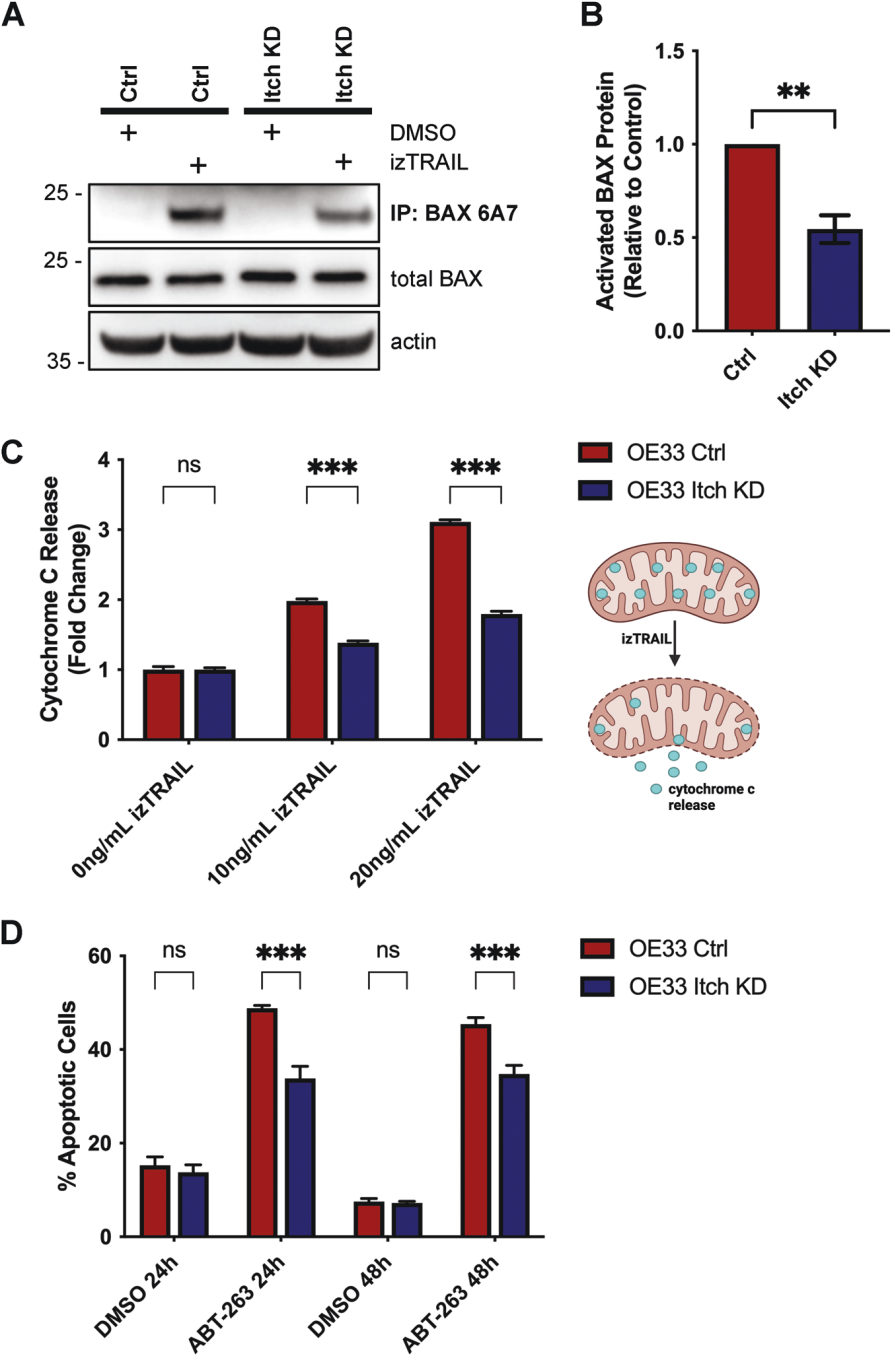
Itch regulates intrinsic apoptosis, Bax activation and cytochrome release. **A** Western blot analysis of an immunoprecipitation experiment using the Bax 6A7 antibody that specifically recognises the activated form of the protein. Total Bax and actin from the OE33 detergent extracts are shown below. Cells were treated with 50 ng/ml izTRAIL for 6 h. **B** Quantification of three independent experiments shown in (**A**). Statistical analysis was performed using Student’s *t* test. **p < 0.01. **C** Flow analysis of cytochrome c release from OE33 control and Itch KD cell lines following stimulation with 10–20 ng/ml izTRAIL for 6 h. In total, 10,000 cells per sample were analysed in three independent experiments. Statistical analysis was performed using 2-way ANOVA. ***p < 0.001. **D** The percentage apoptotic cells were assessed post-treatment with 5 µM Navitoclax (ABT-263) at the indicated time points. Statistical analysis was performed using 2-way ANOVA. ***p < 0.001.

Based on our findings that Itch knockdown results in an accumulation of cholesterol in mitochondria we hypothesised that this altered cholesterol trafficking was the underlying cause of the resistance to TRAIL-mediated apoptosis in the Itch knockdown cells. To test this hypothesis, control cells were treated with the drug U18666A to promote trafficking and accumulation of cholesterol in mitochondria [33]. TRAIL-induced activation of Caspase-8 and Caspase-3/7 in control cells treated with U18666A was inhibited to levels similar to that of Itch knockdown cells (Fig. 5A, B). U18666A used as a single agent did not have any effect on Caspase-8, -3 or PARP cleavage (Fig. 5C, lanes 1–2), but together with TRAIL-treatment inhibited activation of Caspase-8, -3 and cleavage of Bid and PARP (Fig. 5C, lanes 3–4). Quantifications of the data are shown in Fig. 5D–G. The expression of TRAIL-R2 or FLIP were not altered by the treatment. Furthermore, treatment with U18666A reduced the cell surface expression of TRAIL-R2 and TRAIL-R1 (Fig. 5H, I) and increased cholesterol in mitochondria (Fig. 5J), which aligns with the Itch knockdown data. The lipid composition of the mitochondrial outer membrane is important for regulation of Bax/Bak oligomerisation, pore formation and release of cytochrome c. Lipids, such as cholesterol, that increase the stiffness of the membrane attenuate Bax/Bak activation [33, 37]. To investigate the impact of Itch expression on Bax, the protein in its activated conformation was immunoprecipitated with the 6A7 antibody (Fig. 6A). Significantly less active Bax was present in the Itch KD in response to TRAIL treatment (Fig. 6B; *p* < 0.01). To further validate the observation the downstream release of cytochrome c was quantified in a FACS assay. Itch KD cells treated with two concentrations of TRAIL showed strikingly less cytochrome c release compared to the control, which further confirmed the impact of Itch on intrinsic apoptosis (Fig. 6C; *p* < 0.001). Finally, we hypothesised that the effect of an inhibitor targeting anti-apoptotic protein Bcl-2 would be attenuated in the Itch KD. To stimulate the activation of the intrinsic apoptotic pathway directly the Bcl-2 inhibitor ABT-253 (Navitoclax) was used. It is a potent inhibitor of anti-apoptotic Bcl-2 family members (Bcl-2, Bcl-XL, Bcl-W) through its interaction with the BH3-binding groove, causing the release of Bim that activates Bax/Bak which leads to an induction of mitochondrial outer membrane permeabilization and release of cytochrome c. The Itch KD showed a significantly impaired sensitivity to the Bcl-2 inhibitor at 24 h and 48 h (Fig. 6D; *p* < 0.001). Together, these experiments provide evidence for a regulatory role for Itch in regulation of caspase-8 activation through mitochondrial cholesterol content and suggest that Itch mediates TRAIL resistance mainly through regulation of cholesterol trafficking.

### Cisplatin sensitivity is regulated by Itch and mitochondrial cholesterol

**Fig. 7:**
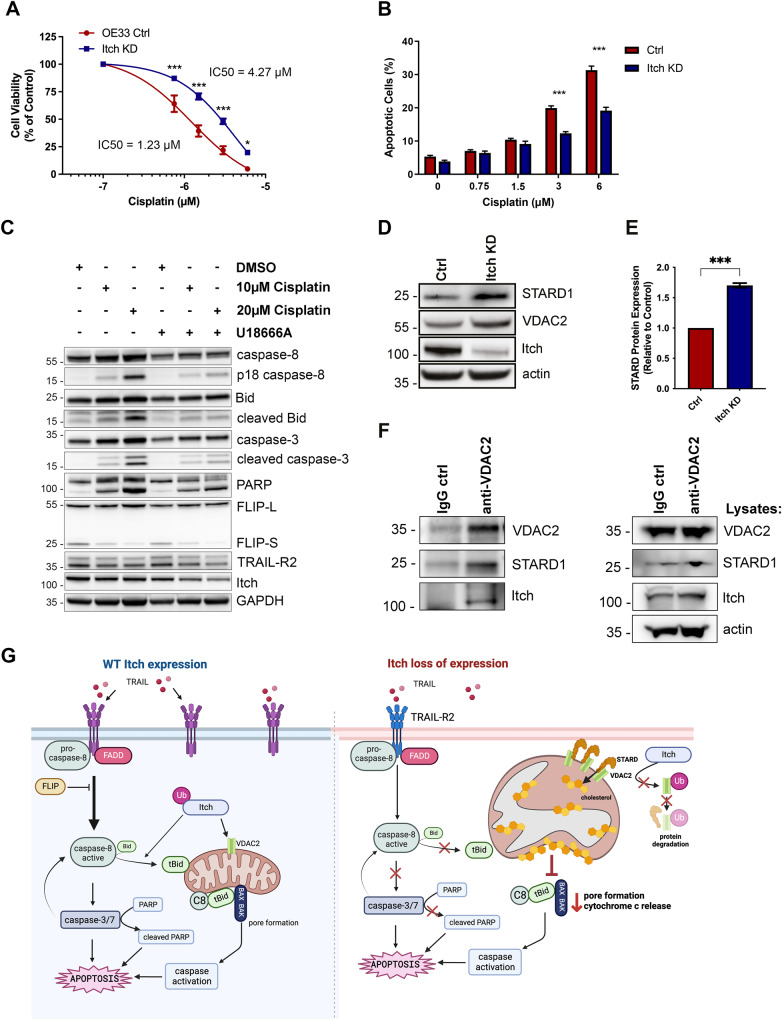
Cisplatin-mediated apoptosis is regulated by Itch expression and mitochondrial cholesterol. **A** Cell viability assay of the Itch KD cell line in response to Cisplatin treatment for 72 h at 0–6 µg/ml. **B** Apoptosis assay using the Itch KD cell line treated with 0–6 µg/ml Cisplatin for 72 h. Data was expressed as a percentage of control. **C** Analysis of apoptotic markers in whole cell lysates from cells treated with Cisplatin in U18666A. **D** Western blot analysis of total expression levels of STARD1 and VDAC2 (dimers) in control and Itch KD OE33 cell lines. Error bars represent the standard error of the mean. Statistical significance was calculated by Student’s *t* test. **E** Quantification of STARD1 band intensity normalised to the loading control in three independent experiments. Statistical analysis was performed using Student’s *t* test, ***p < 0.001. **F** Immunoprecipitation of VDAC2 from OE33 detergent extract. The samples were incubated overnight with a control IgG and the VDAC2 antibody and washed four times. Co-immunoprecipitated proteins were separated by SDS-PAGE electrophoresis and STARD1 and Itch were detected by Western blotting. **G** A schematic image illustrating the suggested model for Itch in regulation of extrinsic and intrinsic apoptosis. A reduction in TRAIL-R at the cell surface impairs downstream apoptotic signalling, which moreover is not further amplified at the mitochondria. Loss of Itch expression results in an increase in mitochondrial cholesterol which impairs membrane fluidity, binding of caspase-8, subsequent activation of Bid and Bax. Together, this results in reduced pore formation in the outer mitochondrial membrane and reduced cytochrome c release. Mechanistically loss of Itch expression results in reduced ubiquitination and degradation of the STARD1/VDAC2 lipid transfer complex that mediates cholesterol import to mitochondria. A stabilisation of STARD1/VDAC2 promotes import of mitochondrial cholesterol which is anti-apoptotic. Statistical significance; *p < 0.05, ***p < 0.001.

Because cleaved Bid synergises and primes cells for apoptosis upon cisplatin treatment, we hypothesised that OE33 Itch KD cells would be resistant to cisplatin treatment due to the reduced Bid levels [38]. Indeed, the Itch KD cell line was significantly more resistant to cisplatin-mediated apoptosis (Fig. 7A, B). A three-fold increase in IC_50_ value in the Itch KD cell line for cisplatin (4.27 µg/ml) compared to the control cell line (1.23 µg/ml) was observed. To investigate the impact of mitochondrial cholesterol on cisplatin-induced apoptosis OE33 cells were treated with UA18666A followed by cisplatin (Fig. 7C). Cisplatin-induced cleavage of caspase-8, -3, Bid and PARP (lanes 1-3) was abrogated by pre-treatment with UA18666A (lanes 4–6). In summary, the data supports a mechanism where Itch and mitochondrial cholesterol trafficking has and important regulatory role in TRAIL- and cisplatin-induced apoptosis. The main protein that shuttles cholesterol to mitochondria is the START domain-containing protein STARD1 that form membrane contact sites between organelles [39]. A clinical study investigating differential gene expression in oesophageal adenocarcinoma in pre- and post-treatment biopsies associated with resistance to chemotherapy [40]. Validations of the hits in siRNA screens reduced the number to seven genes, which included STARD1. Knockdown of STARD1 alone or in combination with cisplatin reduced cell viability in four oesophageal adenocarcinoma cell lines [40]. Thus, low level of STARD1 is a sensitiser to cisplatin treatment in oesophageal adenocarcinoma. We therefore hypothesized that Itch KD cells have increased expression of STARD1 to facilitate cholesterol transfer. Indeed, Western blotting of cell lysates showed elevated levels of STARD1 and its interaction partner VDAC2 (Fig. 7D, E). While STARD1 is not directly regulated by ubiquitination it is stabilised by VDAC2, a protein in the outer mitochondrial membranate that is ubiquitinated by Nedd4 in melanoma cells, a HECT E3 ligase with high sequence homology in the interacting WW domains to Itch [41]. Endogenous VDAC2 from OE33 cells co-immunoprecipitated both STARD1 and Itch (Fig. 7F). Together the data supports a model where Itch negatively regulates the stability of the VDAC2/STARD1 complex at the mitochondrial outer membrane, cholesterol import and activation of apoptosis (Fig. 7G).

## Discussion

Ubiquitination and deubiquitination have emerged as important regulators of death receptor signalling. Particularly in cancer cells the downregulation of pro-apoptotic proteins by ubiquitination have been shown to mediate resistance to cell death. For example, the ubiquitin ligases Cbl and Cbl-b have been associated with TRAIL-mediated apoptosis resistance mechanisms at the plasma membrane where they regulate receptor clustering in lipid rafts and caspase-8 activation [42, 43]. The fact that these ubiquitin ligases regulate both receptor trafficking to cholesterol-rich lipid domains and downstream TRAIL signalling implies that there are additional regulatory mechanisms that affect TRAIL sensitivity. Here we have investigated the function of the ubiquitin ligase Itch in this context. Itch is known to regulate receptor trafficking, intracellular cholesterol synthesis, the stability of the anti-apoptotic protein FLIP and the pro-apoptotic protein tBid [3, 5, 30, 35]. Here, we show for the first time that Itch regulates TRAIL-mediated intrinsic apoptosis through regulation of caspase-8 activation and intracellular cholesterol homoeostasis.

In this study, we observed that knockdown of Itch in OE33 cells results in resistance to TRAIL- and cisplatin-mediated apoptosis. This correlated with reduced TRAIL-R2 at the plasma membrane rather than the expected increased expression of the anti-apoptotic protein FLIP. The protein expression of TRAIL-R2 was not impaired and the localisation of TRAIL-R1 or EGFR were not affected. Together this strongly indicated that Itch-regulated receptor endocytosis that was not enhanced. Regulation of trafficking from the ER/Golgi to the plasma membrane is still an unresolved question for the TRAIL receptors to our knowledge. Interestingly, the exit of other transmembrane proteins that traffic from the biosecretory pathway to cholesterol-rich domains of the plasma membrane, such as the aquaporins, is regulated by cholesterol concentration [44]. The sub-cellular localisation of death receptors do have a significant impact on signalling as shown in a breast cancer cell line where the total protein remained the same, but the cell surface expression of TRAIL-R1 and -R2 was reduced [45]. Further studies are required to dissect these mechanisms. Intracellular signalling through caspase-8 and caspase-3/7 was found to be reduced in cell lines with reduced Itch expression, but caspase-8 cleavage at the DISC was not impaired which indicated that a regulation of mitochondrial-dependent apoptosis was contributing to the phenotype. Furthermore, we observed a significant reduction of t-Bid, which after activation by caspase-8 associates with Bax and Bak and induces mitochondrial membrane pore formation and subsequent cytochrome c release [46]. The sensitivity to TRAIL signalling is regulated by the targeting of t-Bid by the ubiquitin/proteasome system and the mitochondrial membrane composition [3, 20, 33, 47]. A significant accumulation of cholesterol was observed in mitochondria in the Itch KD, along with reduced Bid and PARP cleavage. Treatment with U18666A, a drug that promotes accumulation of cholesterol in mitochondrial membranes, resulted in a loss of caspase-8 activity and cleavage of downstream targets similar to that observed in Itch KD cells. This study provides new insights into the complexity of the intersection between receptor signalling, membrane trafficking and the significance of the ubiquitin E3 ligase Itch in regulating mechanisms of TRAIL-mediated apoptosis.

An important pathway for trafficking of cholesterol to mitochondria occurs through membrane contact sites, and this can be induced by treatment with the drug U18666A [48]. An increase in mitochondrial cholesterol has a strong anti-apoptotic activity due to the inhibition of BAX and BAK oligomerization, which prevents release of cytochrome c and induction of apoptosis [33]. Here we show an impaired BAX activation and cytochrome c release in Itch KD cells. The lipid composition of the mitochondrial outer membrane has an important role in the recruitment and activation of caspase-8 and subsequent cleavage of Bid [32, 34, 49]. A reduced membrane fluidity impairs Bax oligomerisation, resulting in apoptosis resistance [33, 50, 51]. Inhibition of intracellular cholesterol trafficking by the drug U18666A in the HCT116 cell line leads to a mislocalisation of TRAIL-R2 from the plasma membrane to intracellular organelles, loss of caspase-8 activation, PARP cleavage and 5-FU resistance [52]. Chemotherapy resistance is a common issue in cancer therapy that is associated with cholesterol regulation and in particular cholesterol content in the plasma membrane and mitochondria [33, 51, 53–58].

Reprogrammed lipid metabolism is an established hallmark of cancer, and in particular upregulation of the mevalonate pathway [59]. Cholesterol promotes cell proliferation and is regulated by oncogenes (myc, PTEN) and the tumour suppressor p53. The accumulation of cholesterol correlates with a reduction in either a cholesterol exporter or a stabilisation of an importer [41, 51]. Here we propose that Itch-mediated poly-ubiquitination regulates proteins facilitating mitochondrial cholesterol import. A recent clinical study performed a gene set enrichment analysis from oesophageal adenocarcinoma biopsies pre- and post-treatment with chemotherapy [40]. A set of genes from responders to chemotherapy were identified and validated in a siRNA screen. One of 80 hits was STARD1, a cholesterol shuttling protein associated with mitochondria. Knockdown of STARD1 alone or in combination with cisplatin or 5-FU reduce cell viability significantly in four oesophageal cancer cell lines. Oesophageal cancer is not the only cancer type that has been associated with resistance to apoptosis and chemotherapy due to increased mitochondrial cholesterol [53, 60–62]. For example, knockdown of STARD1 in hepatocellular carcinoma sensitises cells to cisplatin and reduces proliferation [51].

Here we report a novel regulatory mechanism for the ubiquitinating enzyme Itch in TRAIL-mediated apoptosis dependent on mitochondrial cholesterol (Fig. 7G). An increase in mitochondrial cholesterol decreases membrane fluidity, increases resistance to apoptosis-inducing agents, impaired caspase-8 activation, Bax activation and cytochrome c release. Trafficking of cholesterol to mitochondria is mainly mediated by STARD1/D3 at ER-mitochondria and late endosome-mitochondria membrane contact sites [63, 64]. Our data show a correlation between STARD1 expression, mitochondrial cholesterol and resistance to apoptosis mediated by TRAIL, cisplatin or Navitoclax. Its overexpression in oesophageal adenocarcinoma treatment-resistant clinical samples and knockdown in the OE33 cell line demonstrates that it regulates the sensitivity to cisplatin and 5-FU [40]. It will be important to further dissect these mechanisms to map the interrelationship between Itch, mitochondrial cholesterol, membrane contact sites, apoptosis and chemotherapy resistance in order to improve patient outcomes.

### Supplementary information

Supplementary figure legends

Supplementary Figure 1

Supplementary Figure 2

Supplementary Figure 3

Original Data File

## Data Availability

The data sets generated during the current study are available from the corresponding author on reasonable request.
