# Supplementary material for: The E3 ubiquitin ligase Itch regulates death receptor and cholesterol trafficking to affect TRAIL-mediated apoptosis
Source: Cell Death Dis. 2024 Jan 12;15(1):40. doi: 10.1038/s41419-023-06417-4 (PMC10786908; doi:10.1038/s41419-023-06417-4)
Supplement: Supplementary file 1 — Supplementary figure legends [file 41419_2023_6417_MOESM1_ESM.docx]

**Supplementary Figure Legends**

**Supplementary Figure 1: Investigation of FLIP expression in a panel of cell lines with Itch knockdown.**

Western blot analysis of basal expression of Itch, FLIP(L) and FLIP(S) in cell lines with a stable shRNA-mediated Itch knockdown (**A**) Lim-1215, (**B**) Colo 320, (**C**) KM12, or a transient siRNA-mediated knockdown of Itch in (**D**) HCT116 and (E) HT29 cells.

**Supplementary Figure 2: Impact of Itch knockdown on TRAIL-R2 cell surface expression in a panel of cell lines.**

FACS analysis of cell surface expression of TRAIL-R2 in **(A)** Lim-1215 Ctrl and Itch KD cell lines, **(B)** Colo 320 Ctrl and Itch KD cell lines, **(C)** KM12 Ctrl and Itch KD cell lines, **(D)** HCT116 cells treated with siRNA, **(E)** HT29 cells treated with siRNA. Bar graph showing the mean fluorescence intensity of cell surface staining of TRAIL-R2 in three independent experiments (n=10,000 cells per experiment). Statistical significance was calculated by Student’s t-test; *p<0.05, **p <0.01, ***p<0.001.

**Supplementary Figure 3: Imaging of free cholesterol and mitochondria in colorectal cancer cell lines upon Itch knockdown.**

Confocal images of HT29 and HT116 cells treated with control or ITCH-targeting siRNA. The cells were stained with Filipin-III (green), a marker of free cholesterol, and an antibody detecting the outer mitochondrial marker TOMM20 (magenta). HT29 cells treated with ITCH siRNA contained more enlarged mitochondria with Filipin-III staining in the lumen (white arrows) compared to control. There was no clear effect on mitochondria upon Itch KD in the HCT116 cell line. Scale bar, 10 µm.

**Supplementary Figure 4: Original full-length Western blots.**
