# Supplementary figures and images for: The E3 ubiquitin ligase Itch regulates death receptor and cholesterol trafficking to affect TRAIL-mediated apoptosis
Source: Cell Death Dis. 2024 Jan 12;15(1):40. doi: 10.1038/s41419-023-06417-4 (PMC10786908; doi:10.1038/s41419-023-06417-4)

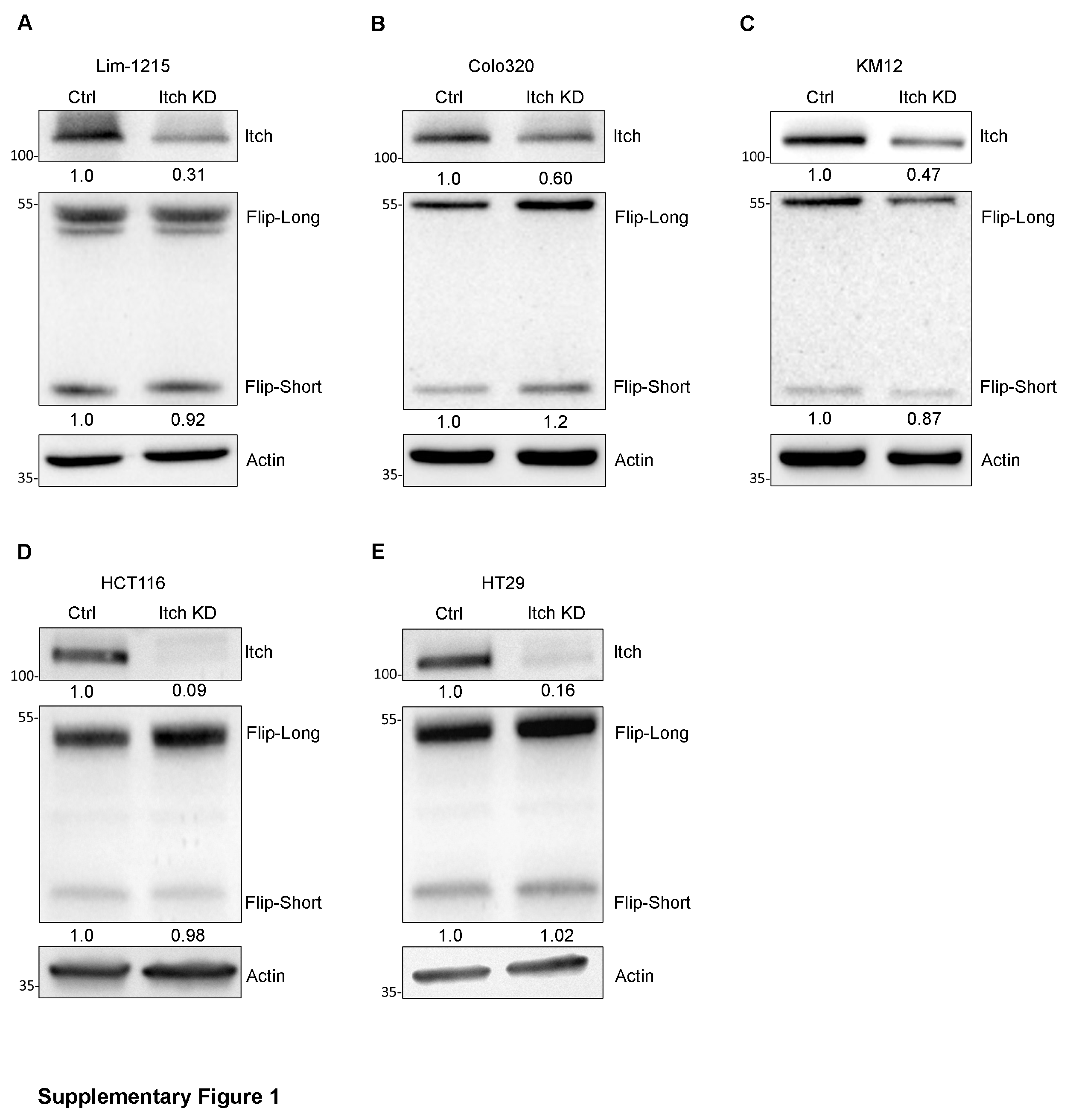

Supplement: Supplementary file 2 — Supplementary Figure 1 [file 41419_2023_6417_MOESM2_ESM.tif]

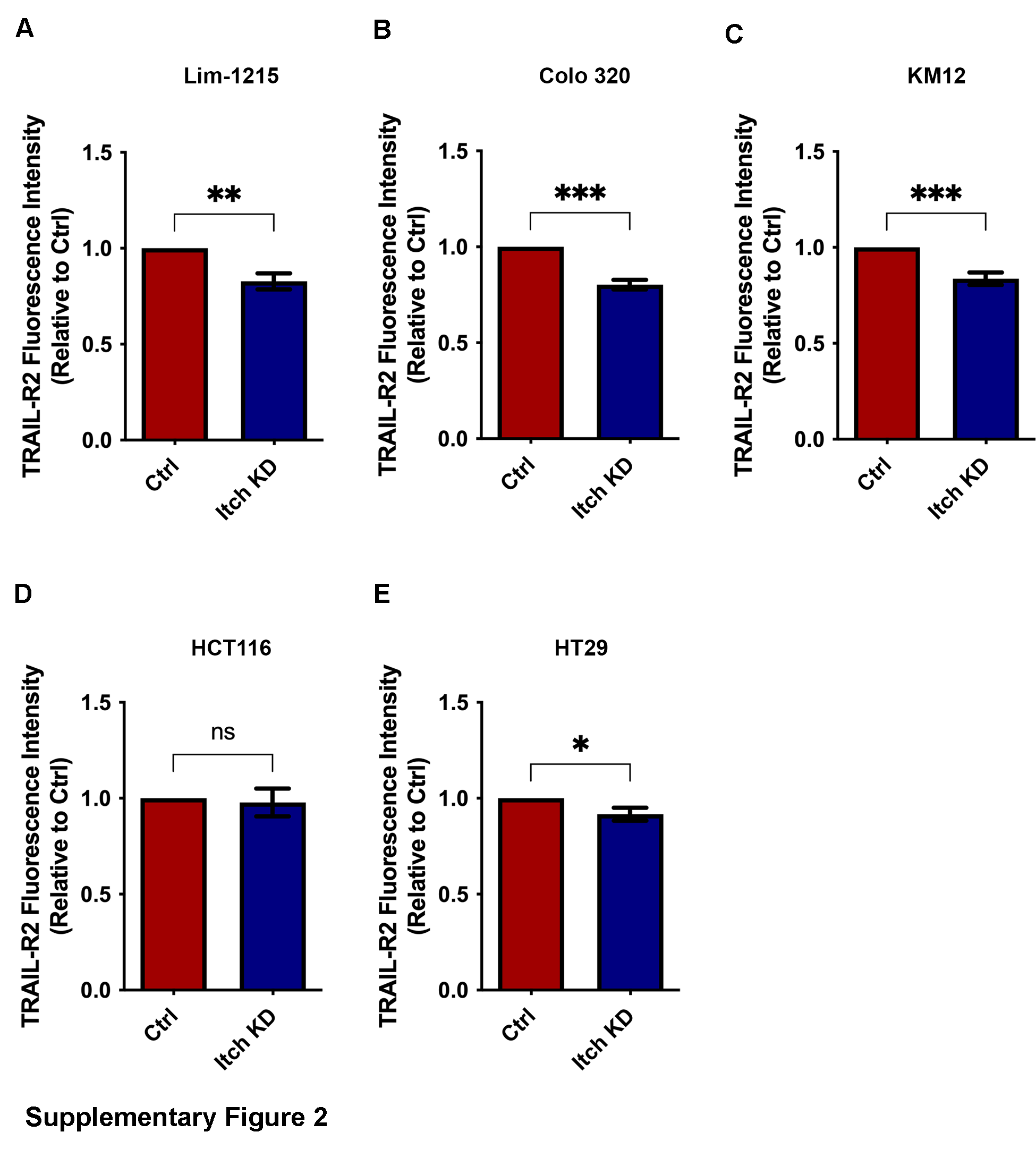

Supplement: Supplementary file 3 — Supplementary Figure 2 [file 41419_2023_6417_MOESM3_ESM.tif]

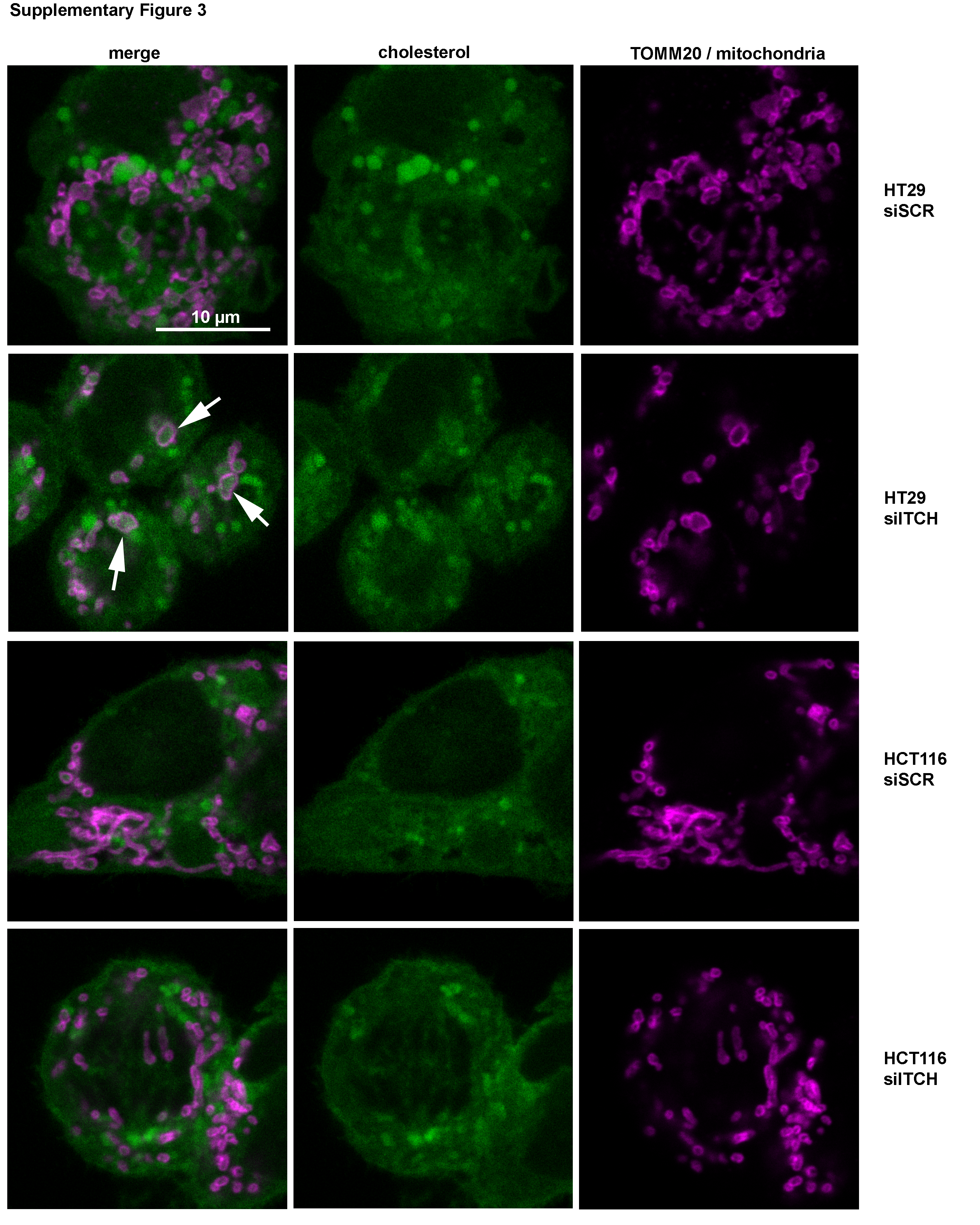

Supplement: Supplementary file 4 — Supplementary Figure 3 [file 41419_2023_6417_MOESM4_ESM.tif]
