# Supplementary material for: The E3 ubiquitin ligase Itch regulates death receptor and cholesterol trafficking to affect TRAIL-mediated apoptosis
Source: Cell Death Dis. 2024 Jan 12;15(1):40. doi: 10.1038/s41419-023-06417-4 (PMC10786908; doi:10.1038/s41419-023-06417-4)

Figure 1A

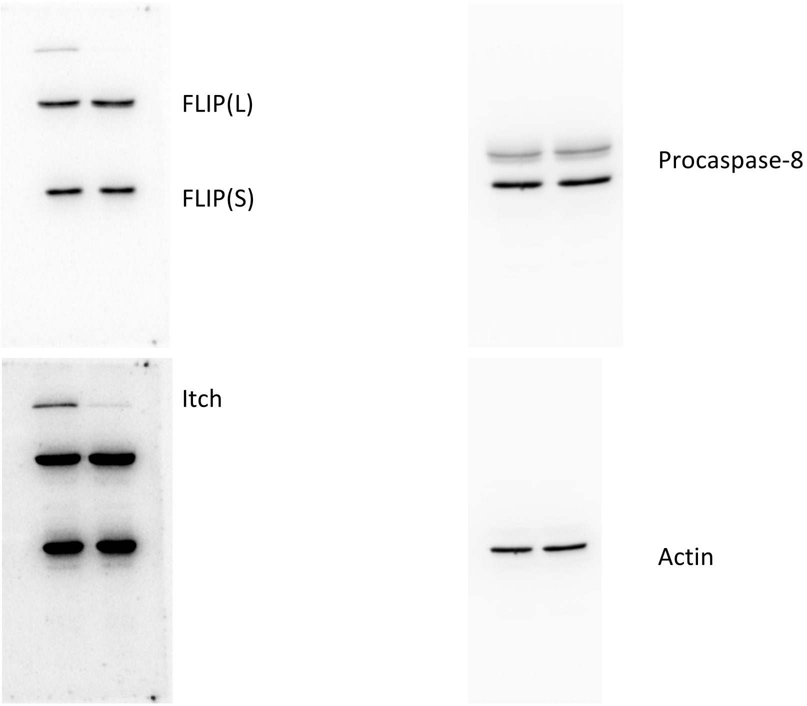

Figure 1H

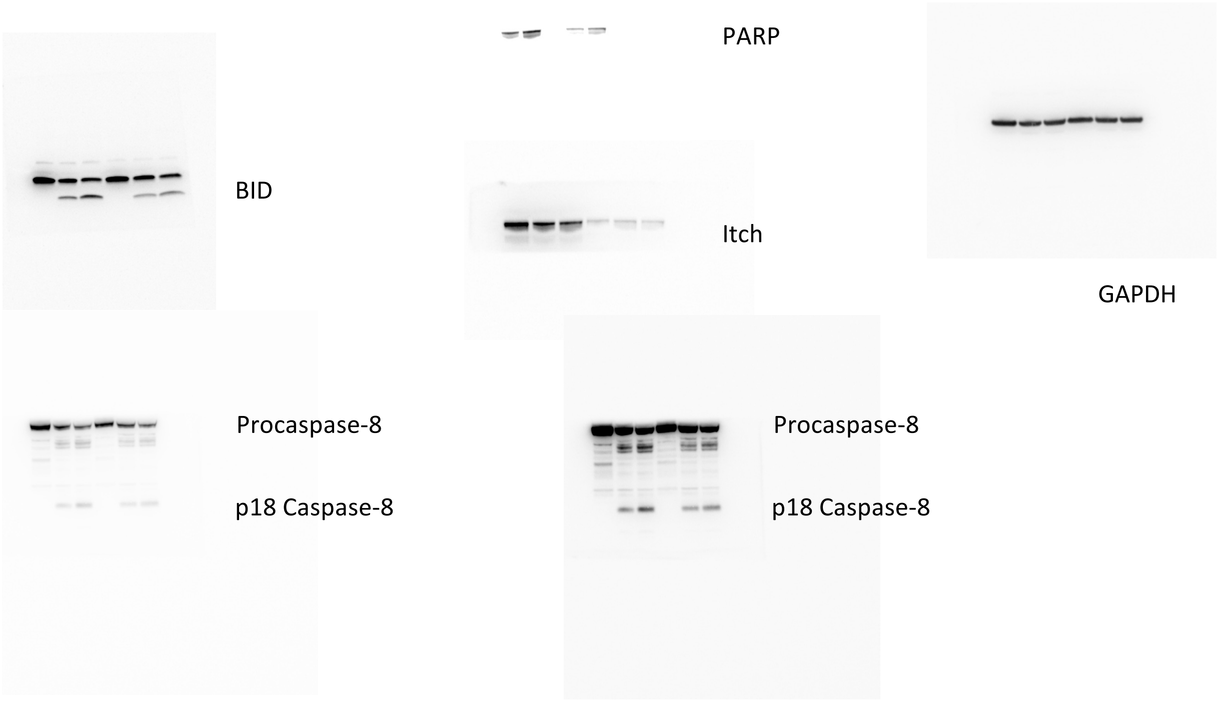

Figure 2F

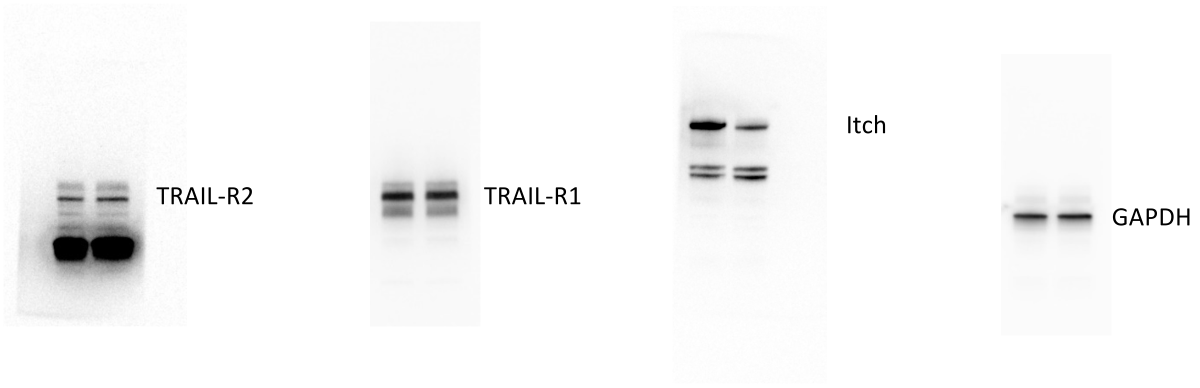

Figure 3A

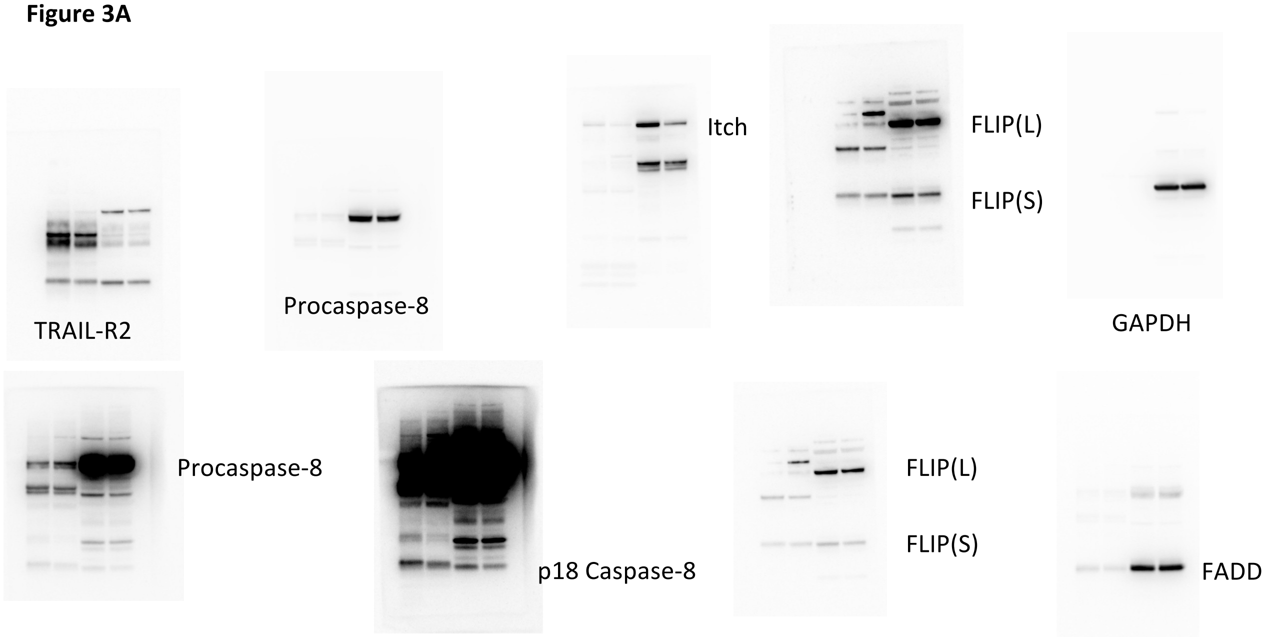

Figure 3C, D

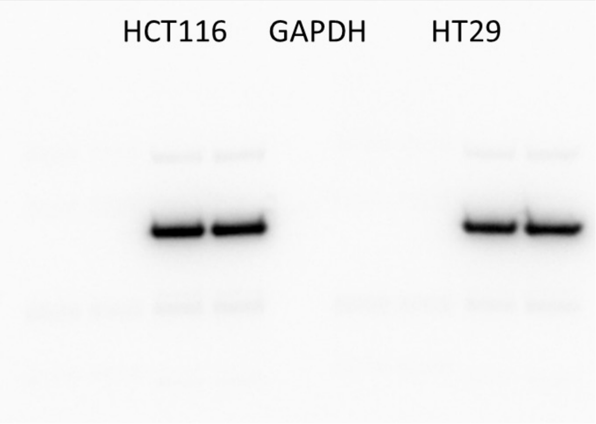
HCT116
GAPDH
HT29

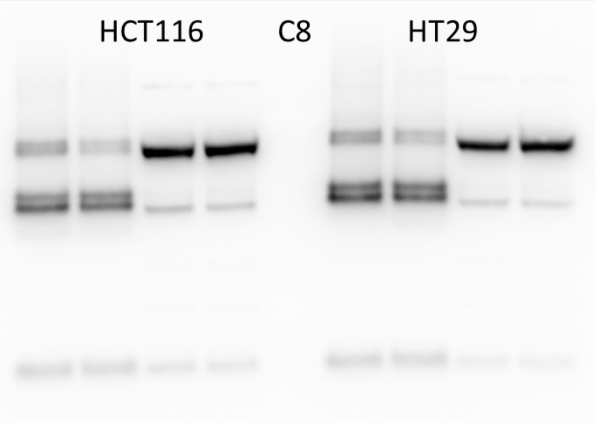
HCT116
C8
HT29

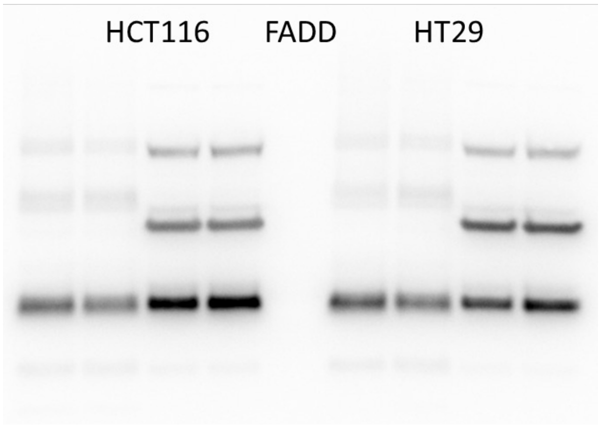
HCT116
FADD
HT29

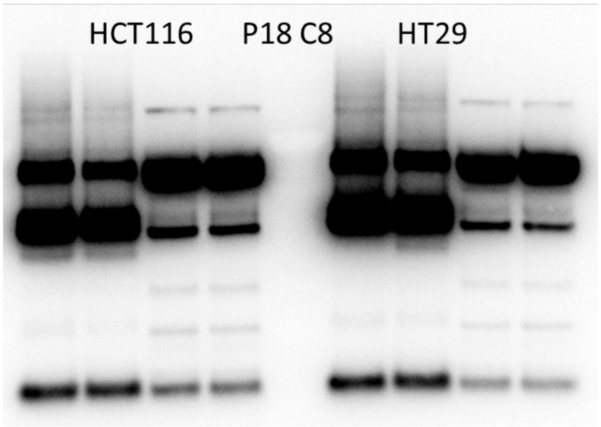
HCT116
P18 C8
HT29

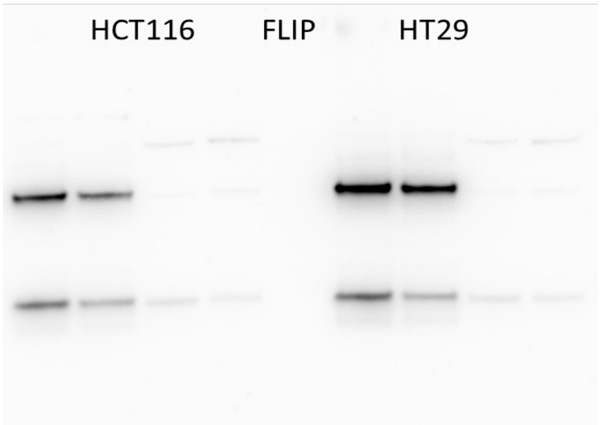
HCT116
FLIP
HT29

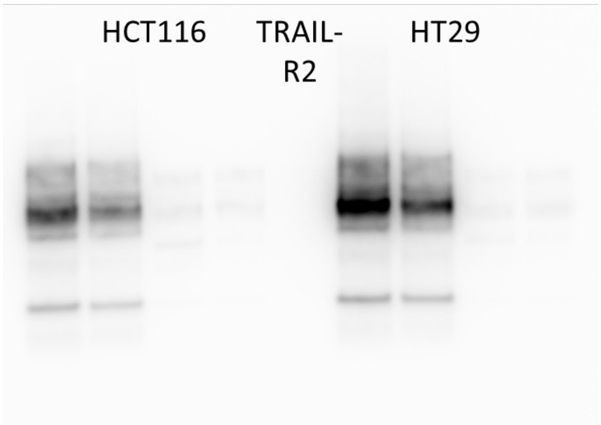
HCT116
TRAIL-
R2
HT29

Figure 4F

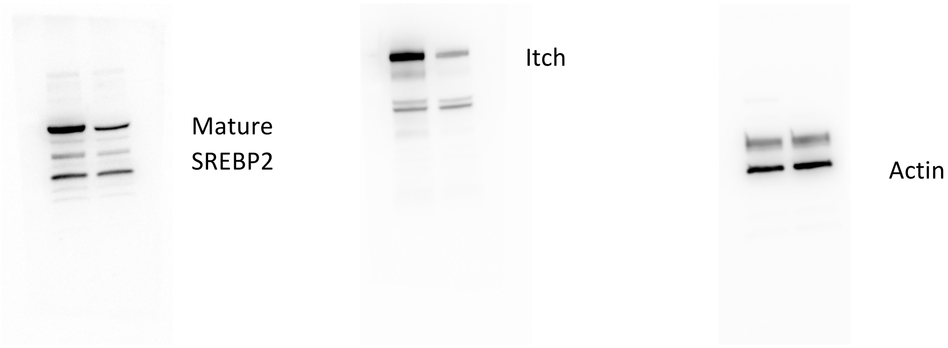

Figure 5C

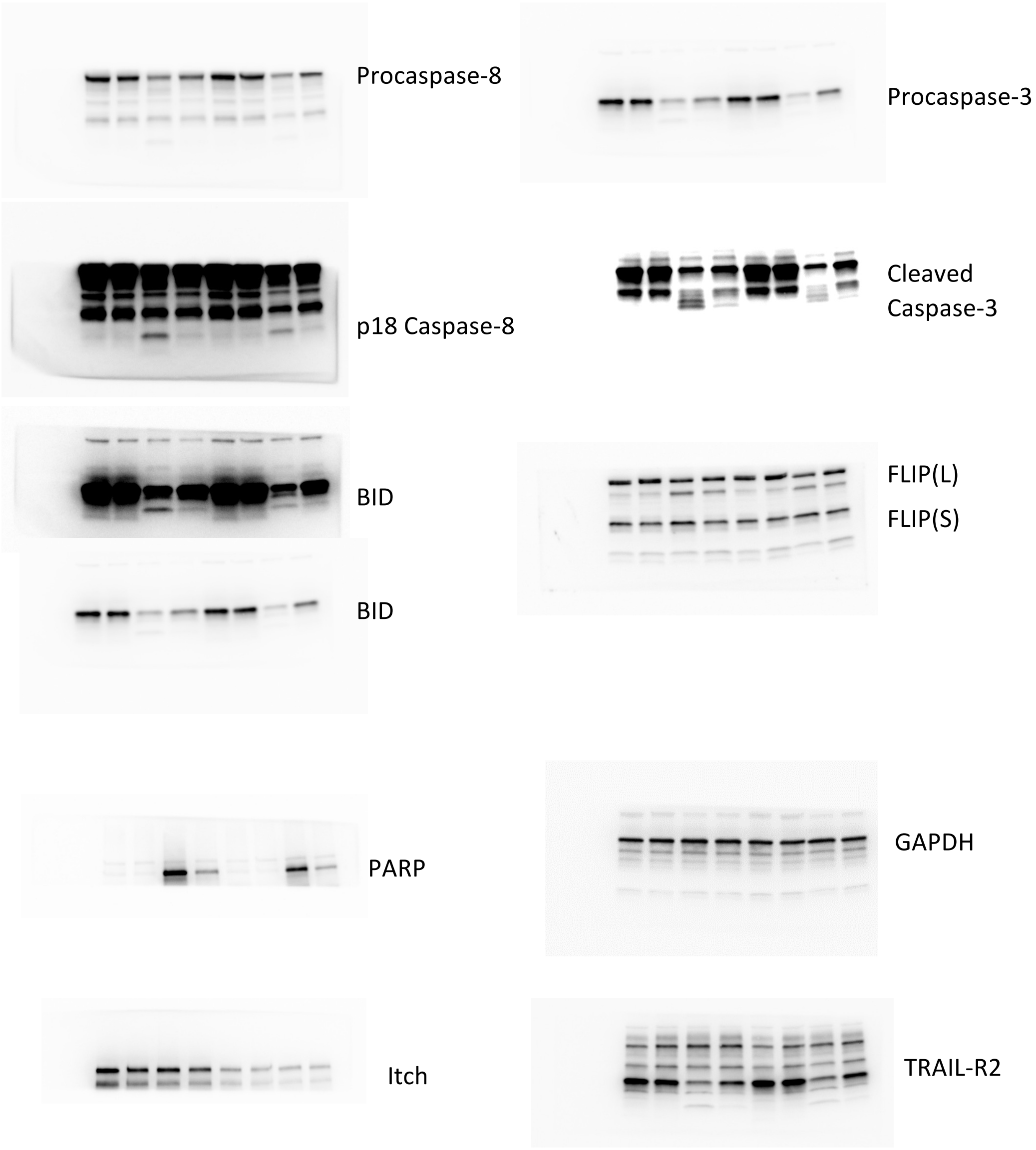

Figure 6A

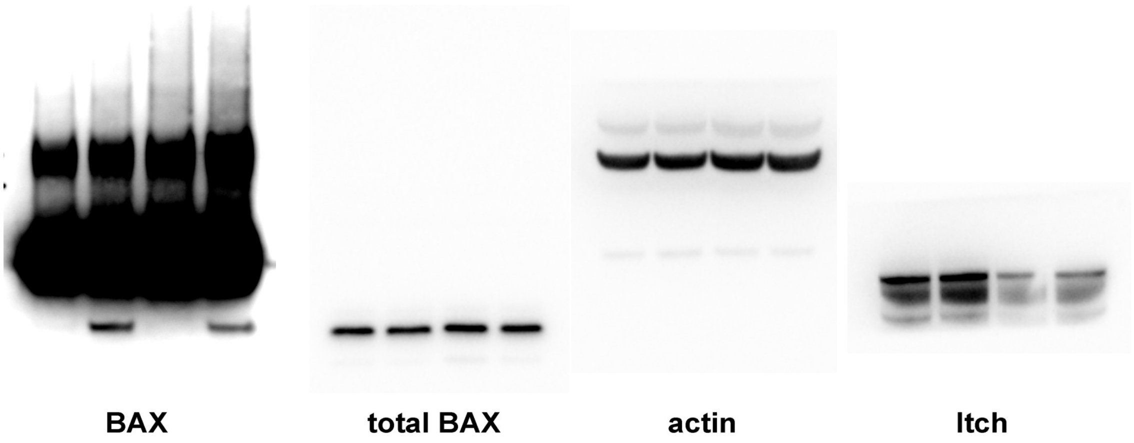

Figure 7C

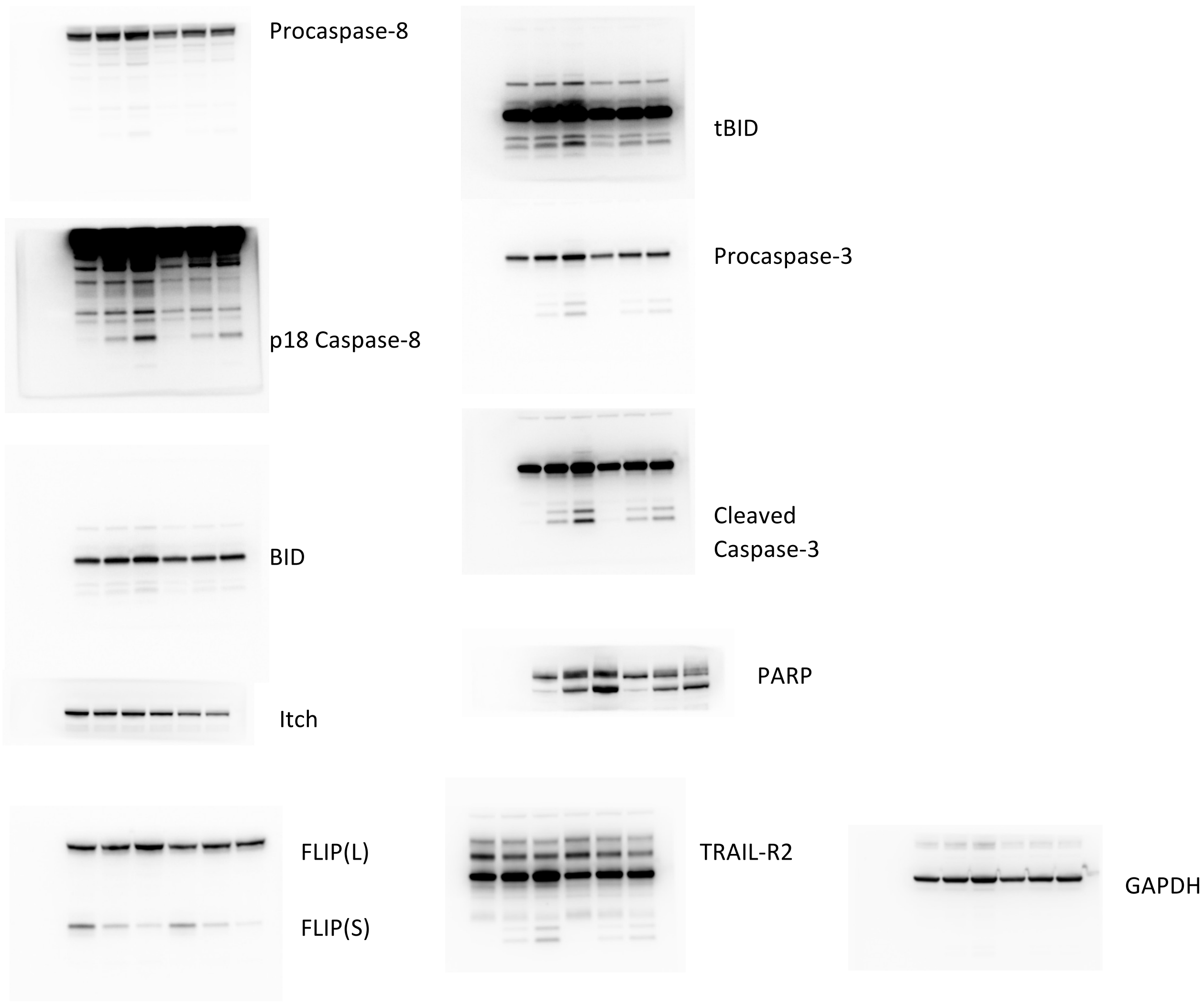

Figure 7D

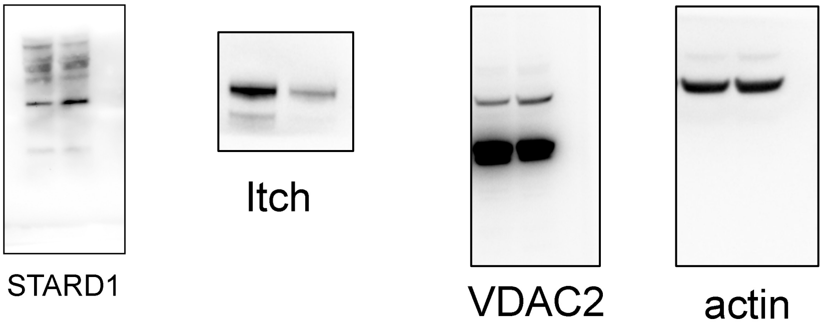

Figure 7F

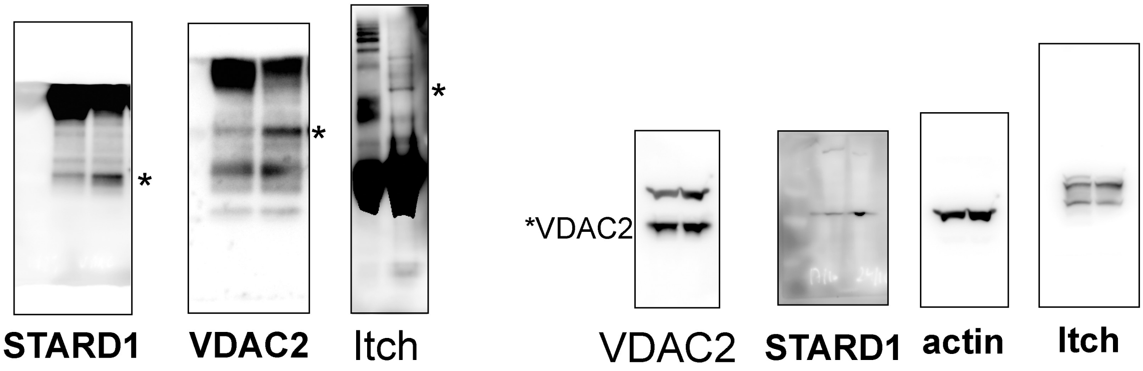

Supplementary Figure 1

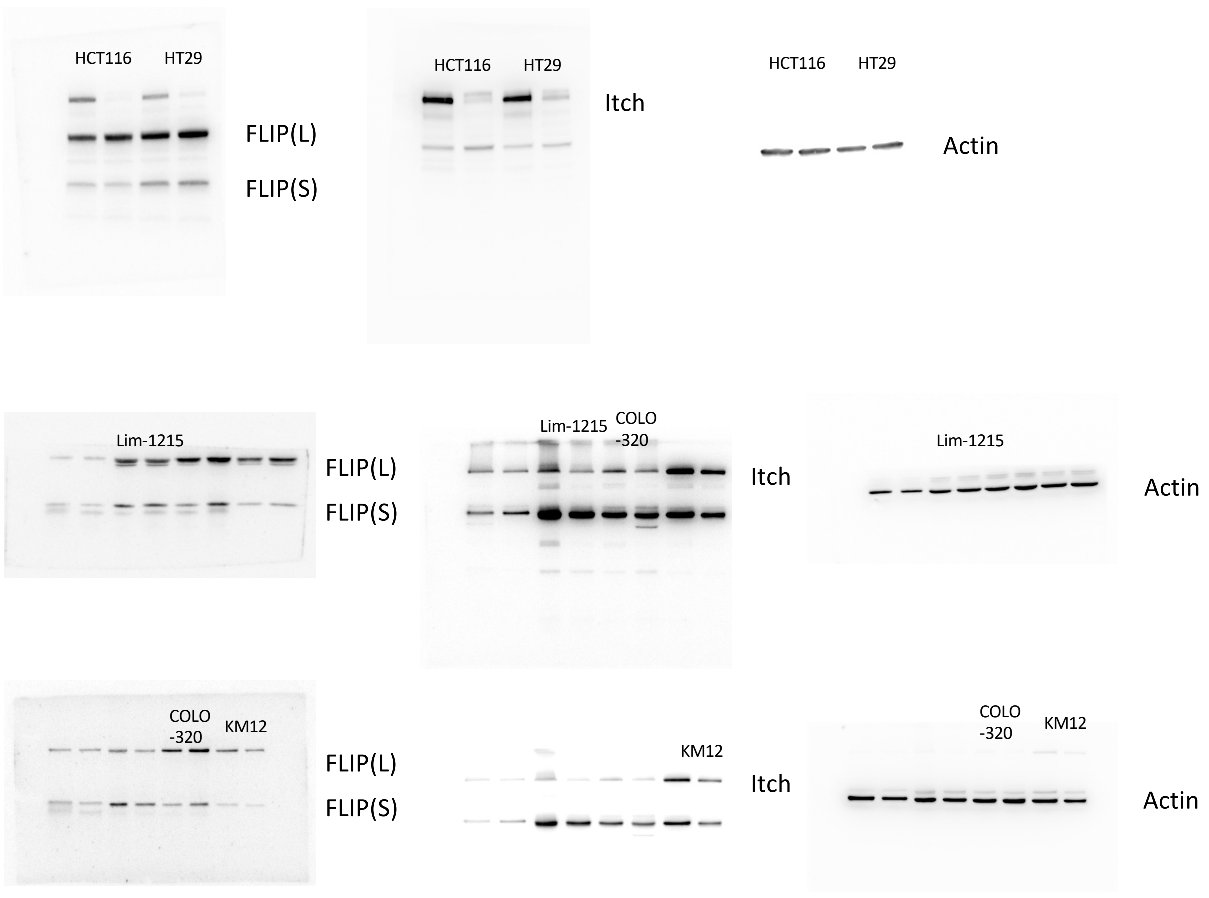

Supplement: Supplementary file 5 — Original Data File [file 41419_2023_6417_MOESM5_ESM.pdf]
